# Seasonal variation in reversal learning reveals greater female cognitive flexibility in African striped mice

**DOI:** 10.1038/s41598-021-99619-9

**Published:** 2021-10-08

**Authors:** Céline Rochais, Hoël Hotte, Neville Pillay

**Affiliations:** 1 School of Animal, Plant and Environmental Sciences, University of the Witwatersrand, Johannesburg, South Africa; 2 ANSES, Plant Health Laboratory – Nematology Unit, Domaine de la Motte Au Vicomte, BP 35327, 35653 Le Rheu Cédex, France

**Keywords:** Ecology, Neuroscience

## Abstract

Cognitive flexibility describes the ability of animals to alter cognitively mediated behaviour in response to changing situational demands, and can vary according to prevailing environemental conditions and individual caracteristics. In the present study, we investigated (1) how learning and reversal learning performance changes between seasons, and (2) how cognitive flexibility is related to sex in a free-living small mammal. We studied 107 African striped mice, *Rhabdomys pumilio*, in an arid semi-desert, 58 during the hot dry summer with low food availability, and 49 during the cold wet winter with higher food availability. We used an escape box task to test for learning and reversal learning performance. We found that learning and reversal learning efficiency varied seasonally by sex: females tested in summer were faster at solving both learning and reversal tasks than males tested in winter. Performance varied within sex: males tested in winter showed faster learning compared to males tested in summer. During reversal learning, females tested in summer were more efficient and solve the task faster compared to females tested in winter. We suggest that seasonal cognitive performance could be related to sex-specific behavioural characteristics of the species, resulting in adaptation for living in harsh environmental conditions.

## Introduction

Cognition enables organisms to process, use and store information gathered from their natural environment^1^ and contribute to reducing uncertainty about important aspects of the environment. There is growing evidence of relationships between cognitive abilities, such as learning, and the fitness benefits in free-living populations^2^. However, cognition is not cost-free, resulting in trade-offs in energy investment between neural or other physiological structures^3^. Therefore, individual variation in cognitive perrformance is expected to closely match the energy demands imposed by the prevailing environmental conditions^4^, an ability that might play an important role in adaptation to new environments, particularly under human-induced rapid environmental change^5^.

Cognitive flexibility is one way to mitigate the need for constant energy investment into cognition, yet there is currently little evidence of links between particular environmental demands and cognitive flexibility^4^. Cognitive flexibility can be defined as “the ability to adapt cognitively driven behaviour in response to changing situational demands”^6^. Because development and maintenance of cognitive processes are energy demanding^7^, energetic constraints in varying environments could induce reversible changes in the structure and functioning of the nervous system, both influencing cognitive performance^8^. However, the environmental conditions under which animals might directly reduce energy investment into cognitive processes (cognitive impairment) and the conditions under which they might keep energetic investment unchanged (cognitive resilience) or improved (cognitive enhancement) remain poorly understood^8^.

The “harsh environment hypothesis” emphasizes the role of variability in food availability in driving cognitive flexibility^9^. Several supporting examples are available in the literature. Comparisons between two distinct populations of black-capped chickadees, *Poecile atricapillus*, showed that birds living at higher altitude and thus experiencing longer and colder winters demonstrated heightened food-hoarding tendencies, more accurate spatial memory (i.e., collection, retention and use of information about the environment to evaluate the relationship between given locations^1^) and higher hippocampal neuron count^10^ but performed worse during a reversal learning task^11^. Woodpecker finches, *Cactospiza pallida*, from an arid, variable habitat performed a single reversal learning faster than birds from a humid, stable habitat^12^. Free living animals could also show adaptive cognitive performance in response to seasonal changes in available energy sources. Spatial learning abilities in the common shrew, *Sorex araneus*, were better in summer than winter, when individuals showed a reduction in brain size^13^. Thus, studying cognitive flexibility according to seasonal changes in food availaibility could help to disentangle different trade-off outcomes between energy saving and cognition^8^.

Reversal learning is a commonly used test of cognitive flexibility^14^, and consists of first training an animal to distinguish between two stimuli, usually through the use of a food incentive (reward). Once the animal is successful at discriminating between the stimuli, the outcome of the reward is reversed and the animal has to inhibit the previously learned response in order to obtain the reward^1^. Reversal learning is ecologically relevant, especially in unpredictable environments, when conditions change rapidly and individuals must be able to inhibit a previously learned behaviour to display a new, more appropriate response. The literature indicates that some species are better at reversal learning and hence displaying cognitive flexibility^15^. For example, pigeons, *Columbia livia*, were more flexible than goldfish, *Carassius auratus*, when given a color discrimination reversal learning task^16^. Several studies also indicate inter-individual variation in cognitive flexibility within species^17^ and suggest the influence of personality^18^ or sex^19^. For example, male and female guppies, *Poecilia reticulata*, learned equally rapidly in a foraging colour discrimination task but females were better at inhibiting the previous response in a reversal task and solved the task twice as fast as males^20^. The authors suggested that sex differences in cognitive flexibility could be related to different roles of males and females in mating competition, mate choice and reproductive behaviour or in social interactions even though it is not known whether differences in cognition that evolved in one context (e.g., reproductive strategies) may affect other behaviours (e.g., foraging)^20^. Although the evolutionary causes are still not clear, previous studies suggested that lower male flexibility and hence, greater male persistence in the same behaviour, may be selected for polygynous species as it helps males to overcome female resistance to mate^20^.

We investigated whether and how discriminating between two spatial stimuli (i.e., learning) and then inhibit the previously learned response (i.e., reversal learning) performance changes between seasons in a free-ranging population of the African striped mouse, *Rhabdomys pumilio.* In our study population, striped mice are diurnal, territorial, polygynous, facultatively group-living, with communal breeding, paternal care and helpers at the nest and mice forage alone during the day^21,22^. *R. pumilio* is an ideal model to study the influence of seasonal variation on cognitive flexibility because it inhabits semi desert areas in southern Africa where it faces marked seasonal changes in food availability^23^. Cognitive performance varied seasonally in striped-mice: mice tested in summer solved a new problem (a proxy of innovation^14^) faster compared to those tested in winter^24^. Furthermore, attention, spatial learning and memory and problem-solving varied seasonally by sex: in summer, males showed a faster attention toward a predator-stimulus, solved more novel problems, but made more errors and took longer in a spatial learning and memory task than males tested during winter^25^. In contrast, the performance of females did not differ seasonally, but their survival was correlated with faster attention to a predator stimulus whereas male survival was correlated with greater spatial memory^26^. Therefore, seasonal cognitive performance in striped mice could be related to sex-specific behavioural characteristics of this species, such as male biased dispersal^27^. Thus, the different sex roles, shaped by natural and/or sexual selection, may also result in sex-specific differences in attention, learning and/or memory, that is, in general cognitive abilities. Evaluating how cognitive flexibility changes according to environmental conditions and sex offer a unique framework to better understand how striped mice cope in challenging environments. Such an approach might also provide a better understanding of the evolutionary causes of different cognitive functions.

We studied sex differences in cognitive flexibility in response to seasonal changes in energy availability. We developed a new escape box protocol based on spatial cues without using food incentive to assess learning and reversal learning performance of free-living striped mice. Using a non-food incentive offers a better assessment of individual variation in cognition because it does not consider a feeding motivation, which is always a challenge in studies in the wild^28^. We tested striped mice during the summer dry and the winter wet seasons. We hypothesized that environemental energy availability (through food availability) changes between seasons will affect cognitive performance such as learning and reversal learning (“ecophysiology of cognition hypothesis”^8^). We predicted that: (1) learning and reversal learning performance will be heightened in summer and follows the harsh environment hypothesis described in birds; (2) seasonal variation in learning and reversal learning will be sex-specific; based on our previous research, we expected that males will show greater spatial learning performance in winter when they disperse whereas females will not differ between seasons but will show overall faster reversal learning performance as found in other species.

## Results

### Seasonal changes in weather, food availability and mice body condition

The weather was hot and dry during summer (temperature: 24.42 ± 0.36 °C; total rainfall: 0.60 mm) and temperatures were lower and rainfall was higher during the winter months (temperature: 13.47 ± 0.45 °C; total rainfall: 39.60 mm; LM: N = 138, F = 368.4, P < 0.001; F = 4.31, P = 0.039, respectively). Food availability for the striped mice increased within the study period from summer (2.35 ± 0.11 food plants/plot) to winter (3.27 ± 0.24 food plants/plot; LM: N_summer plots_ = 48, N_winter plots_ = 48, F = 12.12, P < 0.001).

Body mass and length were greater in winter than summer (body mass: summer: 41.33 ± 1.01 g; winter: 47.17 ± 1.29 g; LMM: N = 107, χ^2^_1_ = 13.44, P < 0.001; Length: summer: 106.21 ± 0.95 mm; winter: 113.91 ± 1.21 mm; LMM: N = 107, χ^2^_1_ = 30.41, P < 0.0001). Males were heavier than females in both seasons (LMM: N = 107, χ^2^_1_ = 38.38, P < 0.0001; summer males: 44.46 ± 1.47 g; summer females: 38.09 ± 1.13 g, t-test: N_males_ = 31, N_females_ = 27, t_96_ = − 3.33, P = 0.006; winter males: 52.20 ± 1.37 g; winter females: 40.29 ± 1.47 g; LMM: N = 44, χ^2^_1_ = 31.54, t-test: N_males_ = 30, N_females_ = 19, t_96_ = − 5.56, P < 0.001). Males were longer than females in both seasons (LMM: N = 107, χ^2^_1_ = 34.55, P < 0.0001; summer: males: 109.21 ± 1.30 mm; females: 102.00 ± 0.99 mm, t-test: N_males_ = 31, N_females_ = 27, t_88_ = − 3.62, P = 0.003; winter: males: 118.12 ± 1.42 mm; females: 108.37 ± 1.43 mm, t-test: N_males_ = 30, N_females_ = 19, t_96_ = -− 4.71, P = 0.0001).

### Seasonal changes in learning and reversal learning

#### Initial task acquisition

All mice (N = 107, Table 1) opened the door for 3 consecutive trials. Some mice did not succeed to open the door at their first attempt and hence needed 4 trials to open doors for 3 consecutive trials (summer: 0/58; winter: 4/49). There was no significant influence of season (GLMM, N = 107, χ^2^_1_ = 0.01, P = 0.985) and sex (GLMM, N = 107, χ^2^_1_ = 0.00, P = 0.999). There was no influence of the season and sex on latencies to open the doors during the 3 consecutive successful trials (LMM, N = 107, 1st trial: season: χ^2^_1_ = 1.40, P = 0.235; sex: χ^2^_1_ = 0.43, P = 0.509; 2nd trial: season: χ^2^_1_ = 2.09, P = 0.148; sex: χ^2^_1_ = 0.21, P = 0.649; 3rd trial: season: χ^2^_1_ = 3.48, P = 0.062; sex: χ^2^_1_ = 0.28, P = 0.597; Supplementary Information: Table 1). However, longer mice showed a greater latency to succeed during their first trial (LMM, N = 107, χ^2^_1_ = 5.38, P = 0.020).

**Table 1:** Sample size for the initial task acquisition, the learning and reversal learning tasks. Mice who were not trapped within 6 days (due to predation or dispersal) were excluded from testing.

Season	Sex	Initial task acquisition	Learning	Reversal learning
Summer	Females	27	24	22
	Males	31	24	23
Total		58	48	45
Winter	Females	19	17	16
	Males	30	22	17
Total		49	39	33

#### Learning task

Overall, we tested 87 of the 107 mice in the learning task since 20 mice were not re-trapped within 6 days after the initial task acquisition phase (Table 1). The learning task was achieved by all subjects within 13.48 ± 0.33 (range: 12–23) trials.

**Figure 1:**
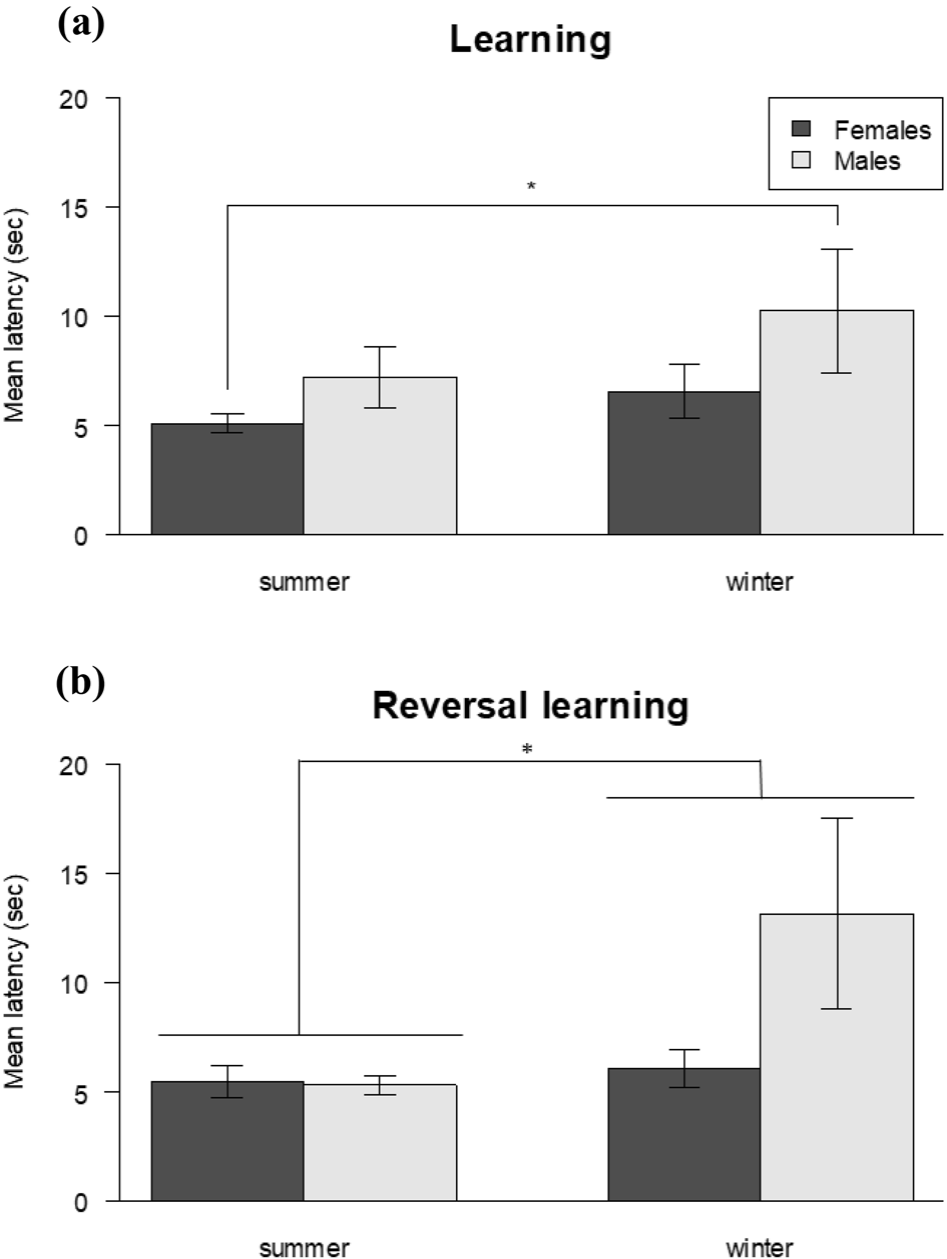
Mean latency ± SEM (in seconds) for all trials needed to reach the learning criterion by season and sex. Males tested in winter showed longer latencies both during the (**a**) the learning task (N_females_ = 41; N_males_ = 46) and (**b**) the reversal learning task (N_females_ = 38; N_males_ = 40). Post hoc t test with Tukey correction, *P ≤ 0.05.

There was no significant influence of season and intrinsic (age, body mass, length) characteristics on the trials to criterion (TTC) and the overall accuracy during the learning task (Table 2). However, there was a significant interaction between season and sex for the mean latency to succeed (LMM, N = 87, χ^2^_1_ = 6.67, P = 0.009). Males tested in winter showed longer latencies compared to females tested in summer (t-test : N_females summer_ = 24, N_males winter_ = 22, t_45_ = − 3.32, P = 0.009; N_females summer_ = 24, N_females winter_ = 17, t_40_ = 0.63, P = 0.921; N_females summer_ = 24, N_males summer_ = 24, t_47_ = − 1.23, P = 0.609; N_females winter_ = 17, N_males summer_ = 24, t_40_ = − 1.10, P = 0.689; N_females winter_ = 17, N_males winter_ = 22, t_40_ = − 1.84, P = 0.269; N_males summer_ = 24, N_males winter_ = 22, t_45_ = − 1.14, P = 0.667; Fig. 1a).

**Table 2:** Results from LMM and GLMM analyses for the influence season, sex, age, body mass, length and group size on the learning performance, group ID is a random factor (bold values P < 0.05).

Predictor variables	R^2^	Estimate	SE	χ^2^	Df	P
	Spatial learning					
**TTC**	0.27					
Season (winter)		0.73	0.61	0.00	1	0.999
Sex (male)		− 0.31	0.33	0.09	1	0.769
Age		− 0.20	0.13	0.13	1	0.717
Body mass		− 0.02	0.02	0.01	1	0.944
Length		0.04	0.03	0.33	1	0.567
Group size		− 0.02	0.01	0.59	1	0.444
Season × sex		0.50	0.49	0.52	1	0.471
**Mean latency**	0.51					
Season (winter)		− 0.40	0.53	0.23	1	0.628
Sex (male)		0.46	0.26	9.82	1	**0.002**
Age		0.12	0.11	1.23	1	0.267
Body mass		− 0.02	0.02	0.59	1	0.441
Length		− 0.03	0.03	1.15	1	0.284
Group size		0.01	0.02	0.28	1	0.594
Season × sex		1.00	0.39	6.67	1	**0.009**
**Accuracy**	0.15					
Season (winter)		0.05	0.15	0.01	1	0.984
Sex (male)		− 0.03	0.08	0.60	1	0.437
Age		0.00	0.03	0.01	1	0.983
Body mass		0.00	0.01	0.01	1	0.939
Length		0.01	0.01	0.82	1	0.364
Group size		0.00	0.00	0.05	1	0.824
Season × sex		− 0.09	0.13	0.54	1	0.461

#### Reversal learning

We tested 78 of the 87 mice in the reversal learning task since 9 mice were not re-trapped within 6 days after the learning task (Table 1). Reversal was attained by all tested subjects within 14.37 ± 0.28 (range: 12–24) trials. There was no significant influence of the season and intrinsic characteristics on the accuracy during the reversal learning task (Table 3). However, there was a significant interaction between season and sex on the trials to criterion (LMM, N = 78, χ^2^_1_ = 4.02, P = 0.045). Females tested in summer tended to need fewer trials to reach the learning criterion compared to females tested in winter (t-test: N_females summer_ = 22, N_females winter_ = 16, t_37_ = − 2.57, P = 0.058; N_females summer_ = 22, N_males summer_ = 23, t_44_ = − 1.15, P = 0.660; N_females summer_ = 22, N_males winter_ = 17, t_38_ = − 0.44, P = 0.971; N_females winter_ = 16, N_males summer_ = 23, t_38_ = 1.26, P = 0.589; N_females winter_ = 16, N_males winter_ = 17, t_32_ = 1.54, P = 0.419; N_males summer_ = 23, N_males winter_ = 17, t_39_ = 0.49, P = 0.959). There was an influence of season on the mean latency to succeed (LMM, N = 78, χ^2^_1_ = 6.67, P = 0.009). Mice tested in winter showed longer latencies compared to those tested in summer (t-test: N_summer_ = 45, N_winter_ = 33, t_39_ = − 3.27, P = 0.011; Fig. 1b).

**Table 3:** Results from LMM and GLMM analysed for the influence season, sex, age, body mass, length and group size on the reversal learning performance, group ID is a random factor (bold values P < 0.05).

Predictor variables	R^2^	Estimate	SE	χ^2^	Df	P
	Reversal learning					
**TTC**	0.28					
Season (winter)		0.23	0.14	2.49	1	0.114
Sex (male)		0.05	0.07	0.39	1	0.530
Age		− 0.02	0.03	0.52	1	0.470
Body mass		0.00	0.01	0.19	1	0.663
Length		− 0.00	0.01	0.35	1	0.555
Group size		0.00	0.00	0.82	1	0.366
Season × sex		− 0.15	0.07	4.02	1	**0.045**
**Mean latency**	0.26					
Season (winter)		0.35	0.35	4.50	1	**0.034**
Sex (male)		0.04	0.21	1.03	1	0.309
Age		− 0.03	0.82	0.09	1	0.752
Body mass		− 0.00	0.02	0.05	1	0.828
Length		0.00	0.02	0.02	1	0.876
Group size		− 0.00	0.01	0.05	1	0.814
Season × sex		0.63	0.33	3.55	1	0.059
**Accuracy**	0.26					
Season (winter)		− 0.17	0.11	2.14	1	0.144
Sex (male)		− 0.03	0.06	0.28	1	0.598
Age		0.02	0.02	0.68	1	0.409
Body mass		0.00	0.01	0.82	1	0.365
Length		− 0.00	0.01	0.17	1	0.676
Group size		− 0.00	0.00	0.40	1	0.525
Season × sex		0.04	0.12	0.12	1	0.724

### Learning curve

**Figure 2:**
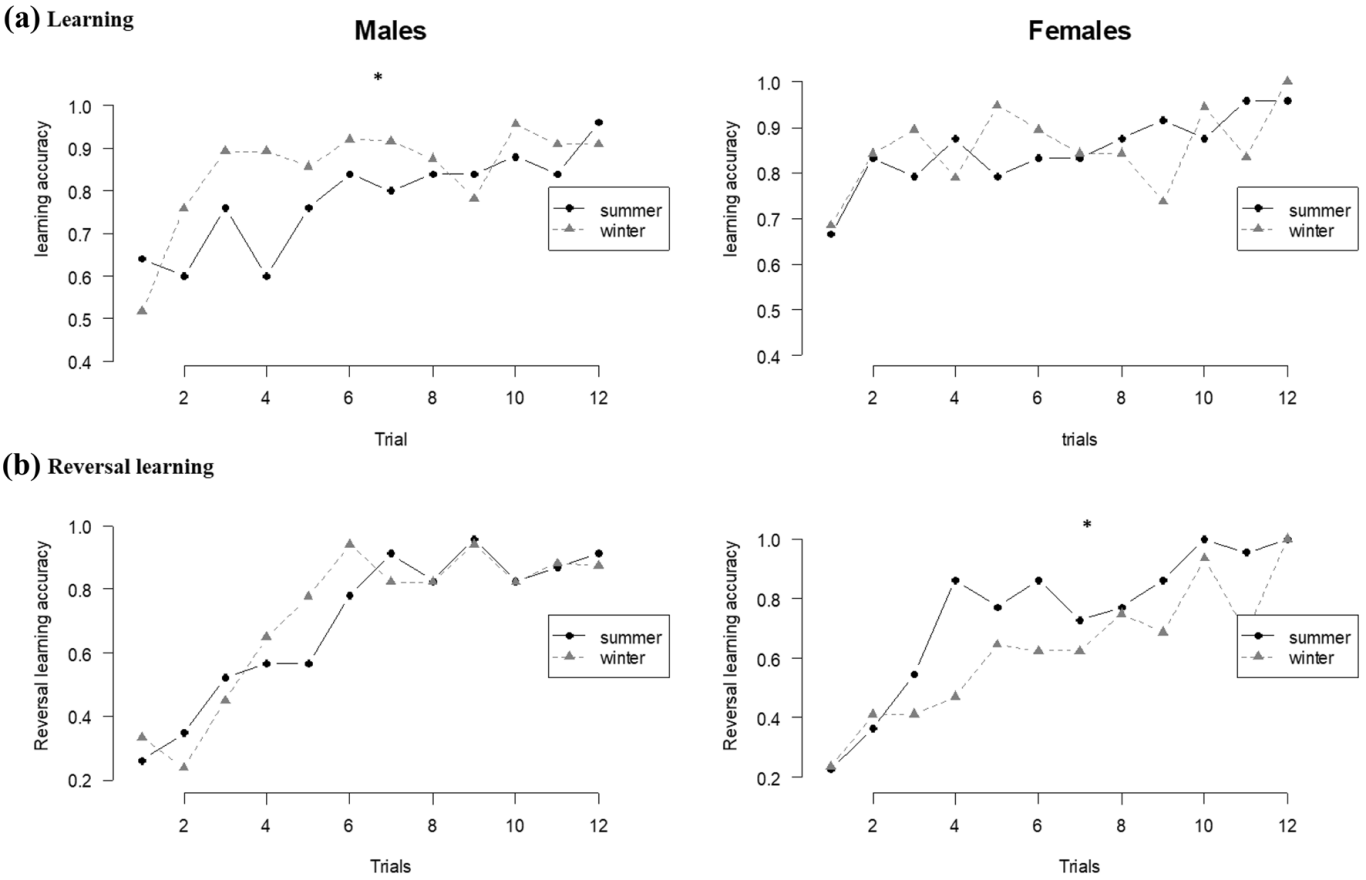
Learning curves in (**a**) the learning (N_females_ = 41; N_males_ = 46) and (**b**) the reversal learning tasks (N_females_ = 38; N_males_ = 40), for the 12 first successive trials. The left panels show males and right panel females. Mice tested in summer are represented with black dots and full black lines and mice tested in winter are represented with grey triangles and gray dashed lines. Post hoc t test with Tukey correction, *P ≤ 0.05.

Learning curves representing accuracy computed for the 12 first successive trials showed a significant effect of trial number (GLMM, N = 87, χ^2^_1_ = 44.59, P < 0.001), which is indicative of learning since the accuracy increased with trial number from the 3rd trial (t-test: Trial 1 *versus* Trial 3 to 12, P < 0.05 for all, Fig. 2a). In addition, there was a significant interaction between season and sex on the response accuracy (GLMM, N = 87, χ^2^_1_ = 4.44, P = 0.035; Fig. 2a). Males tested in summer showed slower improvement in accuracy in learning compared to males tested in winter (t-test: N_males summer_ = 24, N_males winter_ = 22, t_45_ = − 2.97, P = 0.015; N_males summer_ = 24, N_females summer_ = 24, t_47_ = 1.89, P = 0.231; N_males summer_ = 24, N_females winter_ = 17, t_40_ = 0.17, P = 0.998; N_females summer_ = 24, N_females winter_ = 17, t_40_ = − 0.12, P = 0.999; N_females summer_ = 24, N_males winter_ = 22, t_45_ = − 1.13, P = 0.670; N_females winter_ = 17, N_males winter_ = 22, t_40_ = 0.08, P = 0.999; Fig. 2a). For the reversal learning task, there was a significant effect of trial number (GLMM, N = 78, χ^2^_1_ = 150.47, P < 0.001): accuracy increased with trials number from the 3rd trial onwards (t-test: Trial 1 *versus* Trial 3 to 12, P < 0.05 for all, Fig. 2b). There was a significant interaction between season and sex on the response accuracy (GLMM, N = 78, χ^2^_1_ = 6.97, P = 0.008; Fig. 2b). Females tested in summer showed faster improvement in accuracy in reversal learning compared to females tested in winter (t-test: N_females summer_ = 22, N _females winter_ = 16, t_36_ = 2.81, P = 0.025; N_females summer_ = 22, N_males summer_ = 23, t_44_ = 1.86, P = 0.246; N_females summer_ = 22, N_males winter_ = 17, t_38_ = 0.49, P = 0.961; N_females winter_ = 16, N _males summer_ = 23, t_38_ = − 1.11, P = 0.685; N_females winter_ = 16, N_males winter_ = 17, t_32_ = − 2.15, P = 0.138; N_males summer_ = 23, N_males winter_ = 17, t_39_ = − 1.16, P = 0.648; Fig. 2b).

### Speed-accuracy trade-off

**Figure 3:**
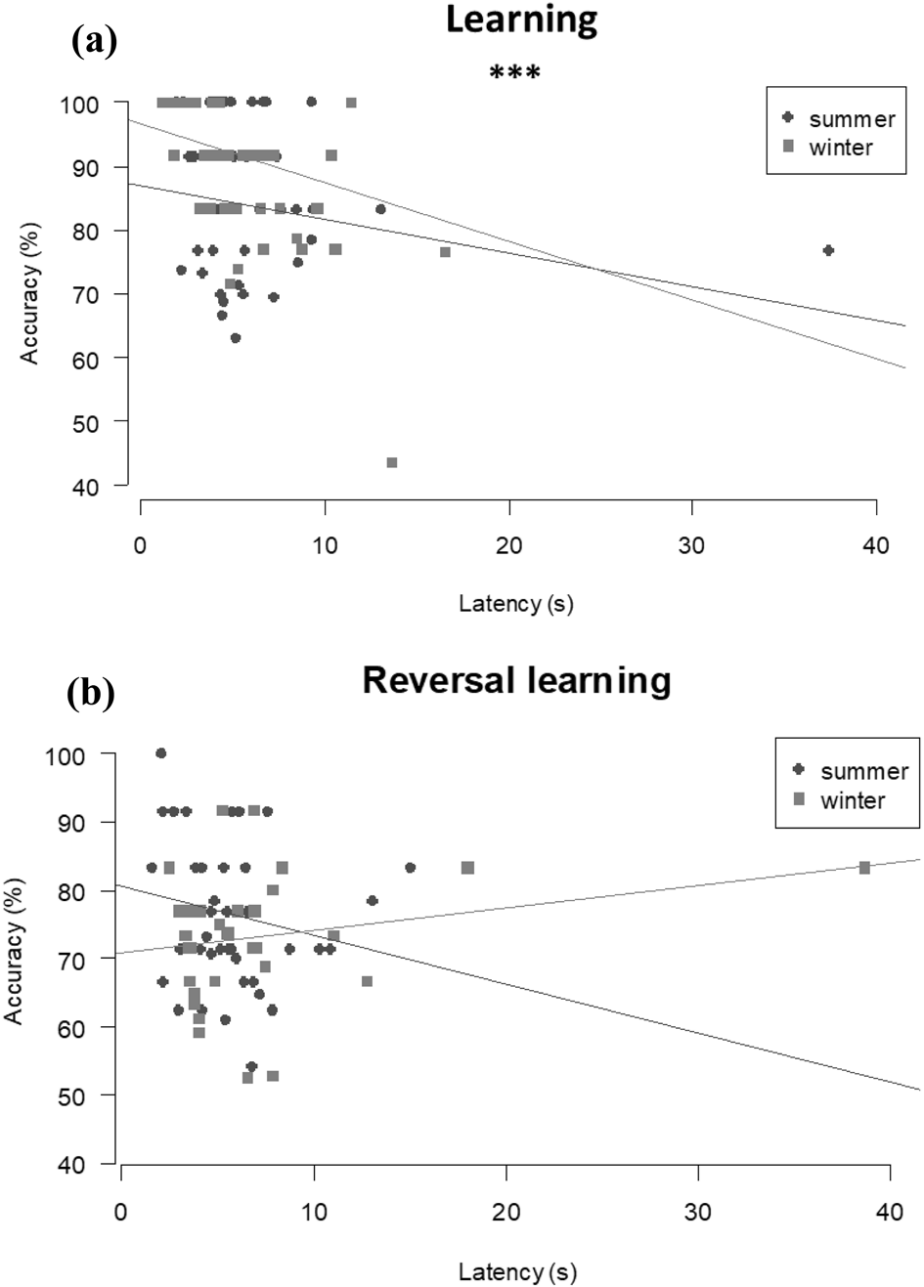
Influence of the season on speed accuracy trade-off measured by the relationship between mean latency (seconds) calculated from all trials during (**a**) the learning (N = 87) task and (**b**) the reversal learning tasks (N = 78); and accuracy measured as the number of correct responses divided by the total number of responses (Spearman rank correlation, ***P < 0.001).

For the learning task, there was a significant influence of the latency (LMM: N = 87, χ^2^_1_ = 8.57, P = 0.004) and the interaction between latency and season (LMM: N = 87, χ^2^_1_ = 7.16, P = 0.009), but no influence of sex (LMM: N = 87, χ^2^_1_ = 1.08, P = 0.301) on accuracy: in winter, mice of both sexes which required more time to solve the task were less accurate (Spearman rank correlation, *summer*: rs = − 0.23, P = 0.111; *winter*: rs = − 0.61, P < 0.001; Fig. 3a). For the reversal learning task, there was no significant influence of the latency (LMM: N = 78, χ^2^_1_ = 0.11, P = 0.741), the interaction between latency and season (LMM: N = 78, χ^2^_1_ = 0.99, P = 0.321) and sex (LMM: N = 78, χ^2^_1_ = 0.17, P = 0.683) on accuracy (Fig. 3b).

## Discussion

We showed that learning and reversal learning performance vary seasonally in a sex-dependent way in a free-living rodent population experiencing seasonal changes in food availability. Learning efficiency, measured with the trial to criterion, as well as accuracy did not differ between seasons and sex. However, males tested in winter showed longer latencies to solve the task compared to females tested in summer. Furthermore, we showed within sex differences in performance according to the season: males tested in winter showed earlier learning curve increase compared to males tested in summer. During the reversal learning task, females tested in summer showed faster reversal learning, needing fewer trials to reach the criterion and showed shorter latencies to solve the task compared to females tested in winter. Thus, in striped mice, there is an inter-relationship between intrinsic characteristics and environemental/ecological influence on cognitive abilities such as learning and reversal learning.

In contrast to previous studies on free-living animals showing increased cognitive performance under harsh conditions, striped mice learning and reversal learning efficiency did not change seasonally, even though summer in our study site was characterized by hot and dry weather with low food availability. This supports the cognitive resilience hypothesis which maintains that cognition is so essential for fitness that animals might keep this energetic investment unchanged, and energy is channelled into cognition even under harsh conditions^29^. Cognitive resilience could rely on specific physiological mechanisms (e.g., brain-centered gluco-regulatory system^30^) protecting the brain against seasonal variation in energy availability and associated changes in hormone secretion^8^. An alternative explanation could be the lower than expected food availability in winter during our study. In other words, cognitive resilience occurred or food availability might not have reached a sufficeint level to induce significant differences in cognitive processes at the population level but could individually influence mice with particular intrinsic (e.g., sex) characteristics.

We showed that learning and reversal learning performance vary seasonally in a sex-dependent way, both between and within the sexes according to the season. Males tested in winter required more time to solve the learning and reversal learning tasks compare to females. This sex effect is unlikely to reflect sex differences in locomotor or motor abilities since the proportion of individuals opening the doors during the initial task acquisition did not differ between males and females. Therefore, this sex difference is likely to reflect the time required for a mouse to “make a decision”. Interestingly, regardless of sex, higher speed (measured as the mean latency) was positively related to the accuracy. Although there is some debate as to whether speed of solving a task is a suitable method to test cognitive proficiency^31,32^, our findings indicate that the mean latency to solve the task is an important metric of cognitive abilities. It appears that striped mice do not suffer of a “speed-accuracy trade-off” where slower individuals might have greater accuracy^33^. Faster striped mice showed greater accuracy, an important adaptation in a prey species which needs to make quick decisions to avoid predators by choosing their route to reach the nest. Males required more time to solve the tasks in winter, indicating that they devoted more time to collect information or were more persistent i.e., time that an animal spent interacting with the task/device^34^ depending on the season. Males disperse in winter and information gathering might be an important adaptation during an important phase of their lives^27^. Alternatively, higher persistence male in winter before the breeding season could be related to the different sex-specific reproductive tactics in this polygynous species^35^. In sum, between sex differences in learning latency according to the season could be related to functional specific behavioural characteristics of this species, such as male biased dispersal^27^ and mating system^35^.

We did not find significant sex differences in the proportion of successful animals or in learning and reversal learning efficiency (TTC and accuracy). Generally, males of several taxa possess better spatial navigation abilities than females^36,37^. Sex differences in spatial abilities may be coupled with instrinsic sex-specific developmental trajectories and subsequent sex differences in reproductive behaviour^38^. For instance, males in some polygynous species have better spatial learning ability compared to females, possibly as a result of greater spatial demands to search for females and consequently have larger home range sizes^38^. In our present study, males did not outperform females in efficiency (TTC and accuracy) during the learning task based on spatial stimuli in each season. These findings could be due to seasonal similarities in activities, such as territorial defense, solitary foraging, facultatively group-living^22^. Furthermore, both sexes were under similar environmental pressure *e.g.,* food availability. Thus, behavioural characteristics do not differ between sexes outside the breeding season and mice were under similar environmental challenges regardless of sex which might explain the absence of spatial learning efficiency difference between males and females.

Interestingly, we found within sex differences according to the season. The faster learning (learning curve) of males in winter compared to summer, concurs with the results of a previous study which showed improved performance within males from summer to winter in spatial learning and memory tests^25^. Improved spatial performance in winter may be adaptive for males that disperse and then breed in spring^39^. For example, spatial memory improved in dispersing male meadow voles, *Microtus pennsylvaniscus,* and deer mice, *Peromyscus maniculatus*^39,40^, during the breeding season relative to the non-breeding season; differences were related to structural modifications in the hippocampus^41^. In striped mice, male dispersal starts in winter, several weeks before reproduction in spring and males will travel distances of up to several kilometres to find new territories^27^. Dispersal will increase demands on spatial learning and memory processing, and thus might explain faster spatial learning improvement in winter as in our study.

During reversal learning, females tested in summer showed faster learning improvement (learning curve) compared to females tested in winter and hence required fewer trials to reach the learning criterion (TTC). This suggests that females potentially respond quicker to environemental changes under harsh conditions^9^. Our results also support previous results on mammals, birds and fish showing that females are more flexible than males^20,42,43^. Several hypothesis have been proposed to explain why females showed greater cognitive flexibility^20^. Males appear to pay a cost for their reduced flexibility, being less ready to modify their behaviour in response to environmental change^35^. Males could show a greater degree of behavioural persistence in the reversal learning task *i.e.,* persistently executing a previously learned behaviour which will result in more errors being made when the contingencies are changed^14^. Such male persistence is supported by our results since males tested in winter had longer latencies to solve the reversal learning task. Thus, males might face trade-offs between the ability to learn new information and the ability to retain old memories^1^. In mammals and birds, sex differences in cognitive flexibility appears to be related to the influence of testosterone level on cholinergic activity in specific brain regions implicated in memory^42,43^. The causes for sex differences in persistence are still not clear, but an adaptive hypothesis suggests that greater male persistence is selected in polygynous species in which males search for reproductively available females^44^. Striped mice in our population are polygynous^21^ which might explain the sex differences in reversal learning in our study in parallel to higher cognitive flexibility for females under harsh conditions.

To our knowledge, this is the first study of reversal learning performance in rodents under natural conditions. However, testing two distinct groups between seasons is an important consideration to test the extent of cognitive flexibility, involving repeat sampling of the same individuals in summer and winter. However, we were mindful of testing the same individual twice since some mice could have remembered the task^25^. Furthermore, it was not possible to test the same individual in both seasons because free-ranging striped mice disperse in early winter^45^ or disappear due to predation. Other studies also reach conclusions about cognitive flexibility between distinct populations e.g., high *versus* low elevation^10^, urban *versus* rural area^4^, humid *versus* arid area^12^ and between seasons (winter *versus* summer^25,46^; breeding *versus* non-breeding^39,40^), and the same individuals were not re-sampled. Finally, it would have been interesting to perform a serial reversal task and paid attention to the improvement in performance over successive reversals^47^ according to the season. However, to perform a serial reversal task with free-ranging animals is challenging, it requires tracking the same individuals in space and time which is difficult with prey species showing dispersal and high predation pressure such as striped mice^45,48^.

In conclusion, our study showed that learning and reversal learning varies according to sex and season in a free-ranging striped mice population. Seasonal differences in cognitive performance could be related to sex-specific behavioural characteristics of the species, such as male biased dispersal^27^ leading to faster spatial learning in winter, and females potentially responding quicker to environemental changes in summer. Such variability in responses indicates cognitive flexibility for males in winter and for females under harsh conditions in summer. This also shows an inter-relationship between intrinsic characteristics and ecological conditions on the influence of cognitive abilities. Thus, the present study creates new areas of investigation on particular environmental demands under which animals might show cognitive impairment, resilience or enhancement and on the relationship with a potential by-product of a sexual selection on other traits. Further studies taking into account the trade-off between cognitive process and energy-demanding physiological processes and their fitness outcomes under harsh seasonal condition are required.

## Materials and methods

### Ethical note

Animal ethical clearance was provided by the University of the Witwatersrand, Johannesburg, South Africa (No. 2018/09/46B). All procedures were in accordance with the ethical standards of the institution or practice at which the studies were conducted. We took care in ensuring the animals welfare throughout the experimental procedures and thereafter. The study was carried out in compliance with the ARRIVE guidelines.

### Study location, period and animals

We conducted our study in Goegap Nature Reserve, South Africa (S 29 41.56, E 18 1.60) during the 2019 dry summer and wet winter seasons. We measured food availability^49^ twice a month and temperature and rainfall daily. Our free-living striped mice population was monitored continuously using behavioural observations, trapping and radiotracking^22^. Mice were trapped using baited metal live traps (26 × 9 × 9 cm) placed under bushes where striped mice were nesting. Trapping sessions were conducted each morning (5 days a week) within the first hour after sunrise. Traps were checked 30 and 60 min after they were set. Evening trapping sessions were conducted 5 days a week within 30 min after sunset^22^. During trapping, mice were sexed and body mass (± 1 g) and length (± 1 mm, from tip of the nose to the anus) were measured. Mice were permanently marked with numbered metal ear tags (National Band and Tag Co., Newport, USA) and with commercial hair dye (Rapido, Pinetown, South Africa) for visual identification.

Striped mice feed on all parts (leaves, flowers and seeds) of annual and periannual plants. We assessed food availability through plant surveys using the Braun-Blanquet method^50^ twice a month on the 1st and the 15th of each month of the year. We recorded the number of food plant species for striped mice as a measure of food availability (palatability is known from behavioural observations^51^), in eight monitoring plots of 4 m2 each that were located within the home ranges of the different social groups^52^.

We assessed learning and reversal learning performance using a novel escape box with a door opening task, in which a test individual could reach its nest as the incentive. We tested 58 mice (27 females and 31 males, originating from 13 different social groups) in summer and 49 other mice (19 females and 30 males, originating from 16 groups including the same 13 groups as in summer) in winter. In order to avoid any carry over effects because of learning, none of the individuals were tested in both seasons. Furthermore, it was not possible to test the same individual in both seasons because striped mice disperse in early winter^45^ or disappear due to predation (*e.g.,* 22 of 55 mice (40%) tested in summer either dispersed or were dead at the beginning of winter).

### Learning and reversal learning tasks

**Figure 4:**
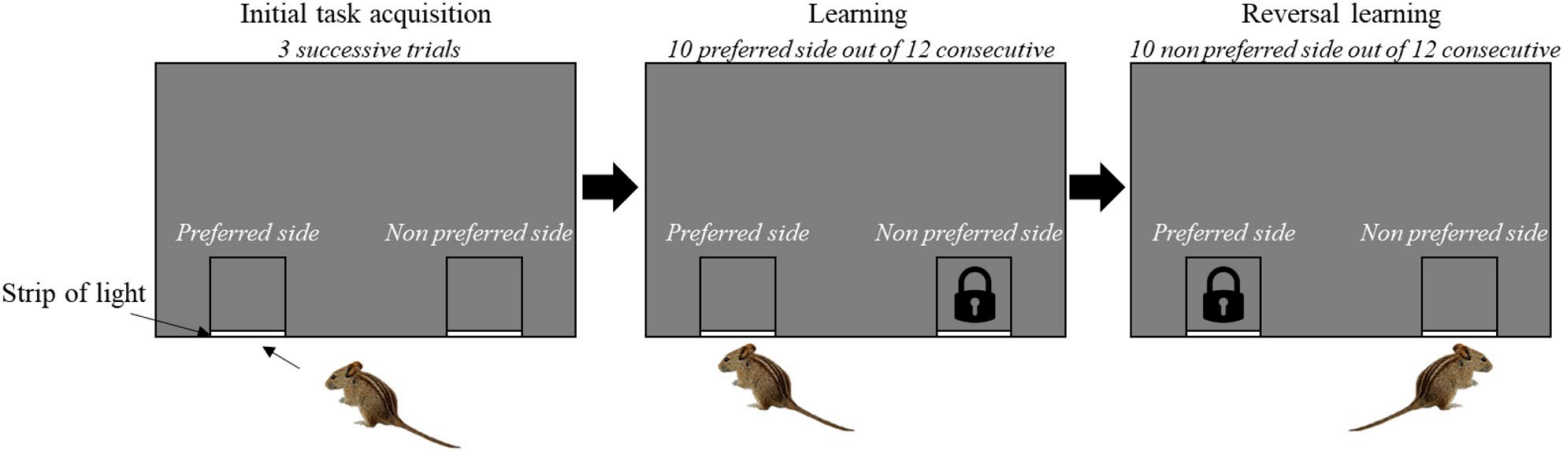
Graphical representation of part of the experimental protocol and device. The mouse has to open one door to escape from the box and return back to its nest. In this example, the mouse chooses the left side (at least 2 left trials out of 3) in the initial task acquisition phase and is then allowed to access the left side (preferred side) during the learning phase whereas the right side door is locked from outside (non-preferred side). After reaching the learning criterion of a total of 10 consecutive correct trials out of 12 during the learning phase, the mouse starts the reversal learning in which it can exit on the right side (non-preferred side) and prevented from exiting the left side (closed door from outside). The mouse had to reach the learning criterion (10 correct responses out of 12 consecutives trials).

Mice were tested directly at their nest in the field in a sequential learning—reversal learning task in which they had to choose between opening a left or right door to reach their nests (Fig. 4). Using a non-food incentive does not test a feeding motivation^28^. After being trapped directly at their nests in both the morning and afternoon (usual trapping sessions), mice were placed by an experimenter (CR) in a black testing box (40 × 30 × 35 cm) equipped with 2 black doors, situated 17 cm apart on the same side of the box and equipped with a transparent perpex lid to prevent mice jumping out of the box (Electronic Supplementary Material). The box was bottomless in order to let mice experience the natural surface. The door system was designed for the mouse to escape and return to its nest (i.e., the incentive) by pushing the door with its head and/or fore paws. The doors were hinged, so that even a light mouse e.g., 25 g, could easily open it. Doors were shorter than the bottom of the box wall, allowing for a strip of external light entering the box and indicating their position (Fig. 4). The escape box was located at 50 cm in front of the nest, with the doors facing the nest. The subject was video recorded using a go pro camera (AEE LYFE, S91B) mounted on the top of the box. Subjects were given a maximum of 5 min to complete the task whereafter those that did not complete the task were released^53^.

Each time a mouse was trapped, its identity was checked by the experimenter and it was placed into the escape box. Each mouse was first tested for 3 times with both doors open and could swing freely (i.e., initial task acquisition^47^) and the door (right or left) it selected 2/3 times was assigned as its preferred one^47^. Once a mouse ended the initial task acquisition (100% of tested mice), it was subjected to the learning task at the next trapping session, where the preferred side was kept open (‘door open’ to reach the nest) and the non-preferred side was locked shut from the outside (‘door closed’ from outside to prevent visual cues of the lock, Fig. 4). An individual was considered to have succeeded at the learning task when it reached the learning criterion of selecting its preferred door at least 10 out of 12 consecutive trials (10/12 correct represents a significant deviation from random binomial probability). A mouse could have as much trials as possible to reach the learning criterion. A trial was defined as each time a mouse was trapped and placed into the apparatus. A mouse was tested only once per trapping session because the incentive was to return to the nest.

During the trapping session after the completion of the learning task, the reversal learning test began where the incentive switched spatially. The initial preferred side was locked shut (‘door closed’) and the previous non-preferred side was rewarded (‘door opened’). Mice had to reach the same learning criterion of at least 10 out of 12 consecutive correct choice trials. Again, a mouse could have as much trials as possible until it reached the 10 correct consecutive trials criterion. If a mouse was not re-trapped within 6 days, it was excluded from the experiment.

### Data collection

We assessed initial task acquisition performance by recording the number of trials needed to open one door for 3 consecutive trials and the latency of each trial as the time required to open the door from the time the mouse was in the escape box. For each experimental task (learning and reversal learning tasks), we assessed the *accuracy of each trial*, by measuring if the response was correct, scored as 1, or incorrect, scored as 0. Response accuracy was computed in sequence for the 12 first trials (regardless of the total number of trials required to attain the 10 consecutive criterion) to assess whether accuracy improved in time^47^ and was used to obtain *learning curves*. Next, we calculated the *trials to criterion *(*TTC*) as the total number of trials (sum of correct and incorrect responses) needed to reach the learning criterion^47^. We computed the *accuracy for all trials* as the number of correct responses divided by the total number of responses. We also measured the latency to open the door for each trial and calculated the *mean latency* in all trials. The TTC and accuracy are measures of performance efficiency and mean latency is a measure of speed efficiency i.e., how fast the mouse took to solve the tasks.

### Statistical analyses

The video-recordings of the tests were scored using the software Kinovea (Kinovea 0.8.15). Scoring was done by the same observer (C.R.) and also by one field assistant who was naïve to the experimental treatment (10% of test sessions, Spearman correlation rank test, N_mice_ = 12, rs = 0.85). All statistics were performed with R v. 3.6.1^54^. The significance level was set at 0.05. Descriptive statistics are reported as means and standard error of mean (SEM). Independence and homogeneity of variances of the models were assessed by inspection of the residuals of the fitted values using the *plotresid* function in *RVAideMemoire* package^55^ and by reporting conditional R^2^ using r.squaredGLMM function of the *MuMln* package^56^. Mixed models were constructed using the lm/lmer or glm/glmer function in *lme4* package^57^ and statistical tests were performed using the Anova function in *car* package^58^. Statistical tests (Likelihood Ratio Test) were performed with loglink function for Gaussian and Poisson distribution and logitlink function for Binomial distribution^58^. Post hoc tests were performed using the *emmeans* function in the *emmeans* package, along with the contrast pairwise comparision function (emmeans, “pairwise”) using t-test with a Tukey correction^59^. We log transformed latencies prior to analysis to achieve improved normality of the model residuals. We checked for outliers and found that the data from some individuals were outliers in one variable and for others in another variable.We maintain that analyses of such experiment should include individual data points as a measure of individual ability and variation, and should not exclude outliers because these account for the population-level variation^60^.

The seasonal changes in weather and food availability were analysed using several linear models (LM) with daily temperature, daily rainfall and bi-monthly food availability measurements as the dependent variables and season as the fixed effect. The seasonal changes in body condition were analysed by using different linear mixed models (LMM) with body mass and length as the dependent variables and season, sex and the interaction between season and sex as fixed effects. We specified group identity as a random factor to account for potential confounding effects of group origin (litter and/or ecology).

When analysing the predictors of initial task training, season, sex, the interaction between season and sex to test sex-specific seasonal variation, age, group size and body condition (body mass and length) were included as fixed effects. We specified group identity as a random factor. We first performed a generalized linear mixed model (GLMM) for Poisson family distribution with the total number of trials needed to successfully open one door for 3 consecutive trials as the dependent variables We performed linear mixed models for the 1st, 2nd and 3rd latencies of successful door opening as the dependent variables.

The influence of season on the learning and reversal learning tasks was analysed using a separate linear mixed model for each task with (i) the trial to criterion (TTC), (ii) the accuracy, or (iii) the mean latency to open the doors as the dependent variables and season, sex, the interaction between season and sex, age, group size and body condition as fixed effect and group ID as a random factor. We analysed predictors of learning curve by fitting GLMMs for Binomial family distribution either for learning accuracy or reversal learning accuracy as dependent variables and trial number, season, sex, the interaction between trial number, season and sex, and age, group size and body condition as fixed effect and mice ID as a random factor.

We analysed the seasonal influence on the speed-accuracy trade-off by testing whether mean latency to open the doors during the learning and reversal learning tasks predicted individual accuracy. We fitted LMMs for each task with the accuracy as a dependent variable and the mean latency, season, sex and the interaction between latency, season and sex as fixed effect and mice ID as a random factor. Post-hoc test were performed with Spearman rank correlation test.

For all previous models, nonsignificant predictors were excluded based on stepwise backward model selection using log-likelihood ratio tests and the Akaike’s Information Criterion (AIC) comparison (‘lrtest’, ‘lmtest’ package^61^).

## Supplementary Information

Supplementary Information 1.

Supplementary Video 1.

Supplementary Information 2.
