# Supplementary material for: Seasonal variation in reversal learning reveals greater female cognitive flexibility in African striped mice
Source: Sci Rep. 2021 Oct 8;11:20061. doi: 10.1038/s41598-021-99619-9 (PMC8501043; doi:10.1038/s41598-021-99619-9)
Supplement: Supplementary file 1 — Supplementary Information 1. [file 41598_2021_99619_MOESM1_ESM.docx]

| **Predictor variables** | | | | **R^2^** | **Estimate** | **SE** | **χ^2^** | **Df** | **P** |
| --- | --- | --- | --- | --- | --- | --- | --- | --- | --- |
|  |  |  | **Initial spatial preference** | | | | | | |
| *Number of trials to 3 consecutive success* | | | | 0.002 |  |  |  |  |  |
| Season (winter) | | | |  | 0.01 | 0.38 | 0.01 | 1 | 0.985 |
| Sex (male) | | | |  | 0.01 | 0.22 | 0.00 | 1 | 0.999 |
| Age | | | |  | 0.01 | 0.08 | 0.02 | 1 | 0.965 |
| Body mass | | | |  | -0.01 | 0.01 | 0.08 | 1 | 0.772 |
| Length | | | |  | 0.01 | 0.01 | 0.05 | 1 | 0.815 |
| Group size | | | |  | -0.00 | 0.00 | 0.01 | 1 | 0.970 |
| Season*sex | | | |  | -0.01 | 0.31 | 0.01 | 1 | 0.986 |
| *Latency 1^st^ trial* | | | | 0.18 |  |  |  |  |  |
| Season (winter) | | | |  | -0.51 | 0.68 | 1.40 | 1 | 0.235 |
| Sex (male) | | | |  | -0.11 | 0.39 | 0.43 | 1 | 0.509 |
| Age | | | |  | 0.03 | 0.15 | 0.03 | 1 | 0.856 |
| Body mass | | | |  | -0.04 | 0.03 | 2.63 | 1 | 0.105 |
| Length | | | |  | 0.08 | 0.03 | 5.38 | 1 | **0.020** |
| Group size | | | |  | -0.02 | 0.01 | 2.56 | 1 | 0.109 |
| Season*sex | | | |  | -0.37 | 0.56 | 0.42 | 1 | 0.515 |
| *Latency 2^nd^ trial* | | | | 0.37 |  |  |  |  |  |
| Season (winter) | | | |  | 0.42 | 0.69 | 2.09 | 1 | 0.148 |
| Sex (male) | | | |  | -0.06 | 0.35 | 0.21 | 1 | 0.649 |
| Age | | | |  | 0.08 | 0.15 | 0.32 | 1 | 0.570 |
| Body mass | | | |  | 0.03 | 0.03 | 1.59 | 1 | 0.206 |
| Length | | | |  | -0.05 | 0.03 | 2.19 | 1 | 0.138 |
| Group size | | | |  | -0.01 | 0.02 | 0.68 | 1 | 0.409 |
| Season*sex | | | |  | 0.71 | 0.51 | 1.95 | 1 | 0.162 |
| *Latency 3^rd^ trial* | | | | 0.19 |  |  |  |  |  |
| Season (winter) | | | |  | 0.73 | 0.61 | 3.48 | 1 | 0.062 |
| Sex (male) | | | |  | -0.31 | 0.34 | 0.28 | 1 | 0.597 |
| Age | | | |  | -0.20 | 0.13 | 2.28 | 1 | 0.131 |
| Body mass | | | |  | -0.02 | 0.02 | 1.01 | 1 | 0.315 |
| Length | | | |  | 0.01 | 0.03 | 2.03 | 1 | 0.154 |
| Group size | | | |  | -0.02 | 0.01 | 1.53 | 1 | 0.215 |
| Season*sex | | | |  | 0.50 | 0.49 | 1.05 | 1 | 0.306 |

Table 1: Results from LMM and GLMM analyses for the influence of the season, sex, age, body mass, length and group size on the initial task acquisition, group ID is a random factor (bold values **P < 0.05**).

Supplementary information 2:

Video caption: A mouse in the escape box apparatus during the initial task acquisition.
